# Determination of Volatile Water Pollutants Using Cross-Linked Polymeric Ionic Liquid as Solid Phase Micro-Extraction Coatings

**DOI:** 10.3390/polym12020292

**Published:** 2020-02-02

**Authors:** Yuan Tian, Xilan Feng, Yuping Zhang, Quan Yu, Xiaohao Wang, Mengkui Tian

**Affiliations:** 1 Division of Advanced Manufacturing, Tsinghua Shenzhen International Graduate School, Shenzhen 518055, China; tianyuan1700@163.com; 2 School of Chemistry and Chemical Engineering, Henan Institute of Science and Technology, Xinxiang 453003, China; fengxilan1964@163.com (X.F.); beijing2008zyp@163.com (Y.Z.); tmk1964@163.com (M.T.)

**Keywords:** solid phase micro-extraction, polyionic liquids, cross-linker, in-mold coating, polycyclic aromatic hydrocarbons, river water

## Abstract

Ionic liquids found a wide application in catalysis and extraction due to their unique properties. Herein, ethylene glycol dimethacrylate as the cross-linker and 1-vinyl-3- butylimidazolium tetrafluoroborate as functional monomer via thermally initiated free-radical polymerization was prepared as a novel copolymer solid phase micro-extraction (SPME) coating. A surface modified stainless-steel wire was implemented as the substrate. Factors affecting the extraction performances of the copolymer, including the molar ratio of monomers to cross-linkers, the amount of porogen agent, and polymerization time were evaluated and optimized. To evaluate the extraction performance, five commonly seen polycyclic aromatic hydrocarbons (PAHs) were taken as the analytical targets. The potential factors affecting extraction efficiency were optimized. The as-prepared SPME device, coupled with gas chromatography, was successfully applied for the determination of PAHs in water samples. The wide linear range, low detection limit, good reproducibility, selectivity, and excellent thermal stability indicate the promising application of the newly developed SPME fiber in environmental monitoring as well as in other samples having complex matrices.

## 1. Introduction

Environmental protection became an issue and gradually stepped into the public’s vision as a result of the extensive management of industrialization and urbanization in the past century. The concept of green chemistry, first proposed by Kletz, Nameroff, and Badami [1,2,3], with the initial goal of minimizing waste generation, gradually evolved into the development of environment-friendly production and pollution prevention. Necessary monitoring and detection methods should be developed to facilitate the governing, of which the applicable methods should also be green in order to alleviate the pollution pressure. PAHs (polycyclic aromatic compounds) derive from the incomplete combustion of coal, wood, gasoline, wood and agricultural waste, which are widely present in soils, rivers, atmosphere, and foods [4,5,6,7]. Long-term exposure under PAHs may lead to cancer [8,9,10]. Environmental pollutants have the characteristic of trace amounts and a wide range of coverage. Analyzing or detecting these complicated environmental samples requires extraction, purification, and concentration steps that are conventionally redundant and consume a lot of organic solvents, which contradict the motivation of protecting the environment.

Solid phase micro-extraction (SPME) integrates multiple sample processes and saves solvents, time, and labor in the sample pretreatment. It was introduced in the 1990s [11] and soon penetrated into various fields due to its simplicity, low-cost, ease of fabrication, fast analysis, no or minimal use of solvents, and high sensitivity when used in combination with HPLC (high-performance liquid chromatography) or GC (gas chromatography). As a typical fiber format, the function of extraction is realized by coating solid phase adsorbent materials on the fiber substrate. While several coating fibers like PDMS (polydimethylsiloxane) and PA (polyacrylate) are commercially available, their performance is not always satisfactory because of their ease of swelling or peeling off in organic solvents or breakage when using quartz fiber as the coating substrate [12,13]. Therefore, developing a novel fiber with excellent cost-effectiveness, high chemical and thermal stability, good mechanical strength, selectivity, and ease of preparation become the main drives behind the prosperous SPME analysis. Currently, new adsorbent materials, including carbon nanomaterials [14], organoclays [15], metal-organic frameworks (MOFs) [16], molecular imprinted polymers [17], ionic liquids (ILs) [18], and polymeric ionic liquids(PILs) [19] have been proposed as the SPME fiber coatings. Meanwhile, coating methods, such as physical coating [20], sol-gel technique [21], electrodeposition [22], and in-mold approaches [23] have also been developed.

Among these materials, ILs and PILs have attracted a lot of attention. Their unique physicochemical properties, including high chemical and thermal stability, low volatility, as well as the interactions with various analytes through solvation mechanisms. ILs are a type of liquid that is comprised entirely of ions. They are formed via the electrostatic forces between the anion and cation moieties and can be finely tuned with different substituents, enabling their performance to be designed at the molecular level to meet any specific needs [24]. For example, by imparting the hydrophobic part of IL in the extraction media, they may possess a specifically high affinity towards non-polar substituents. IL-based solid phase coatings were first introduced by Liu et al. in 2005 [18] to analyze benzene, toluene, ethyl benzene, and xylenes. In this report, the fiber had to be re-coated with IL after each use, reducing the inherent convenience of the SPME method. To overcome this challenge, while maintaining the unique properties of ILs, Zhao et al. prepared SPME fibers by polymerizing ionic liquids; the prepared PIL coating exhibited long lifetimes and good reproducibility [19]. Since then, extensive studies have been made in the application of PIL as an SPME coating [25,26,27,28].

While PIL-based SPME coatings selectivity can be flexibly adjusted, exposure to high concentrations of organic solvents or high temperature may lead to swelling or peeling, reducing their robustness [29]. Generally, the addition of a cross-linker to the pre-polymerization solution would decrease the solubility of the final copolymer products in organic solvents while enhancing its thermal, chemical, and mechanical stability [30,31]. Among these ionic liquid copolymers, the most commonly used cross-linker is another ionic liquid [32,33,34,35]. Only a few of them, like ethylene dimethacrylate [36] and ethylene glycol dimethacrylate [31] were reported.

Ionic liquids have received attention in the extraction techniques since they are thermally stable, compatible with various organic solvents, easy to be tailored, and capable of conducting anion exchange. The commonly used imidazole ionic liquids typically contain X^−^, PF_6_^−^, BF_4_^−^, and NTf_2_^−^. X- based ionic liquid coating has the drawback of poor robustness, and ease of absorbing water and swelling under high temperature [37,38]. Therefore, the cheap imidazole tetrafluoroborate or hexafluorophosphate were most used in the preparation of the extraction coating.

Ethylene glycol dimethacrylate (EGDMA) is a commonly used cross-linker with dual-functional groups, the double bond on its side chain has high reactivity and can compete to react with the double bond in monomers during the polymerization process. The degree of cross-linking is thus increased as the unreacted double bonds on the branches of the bulk polymer may react with each other in itself [39]. Cross-linking is frequently adopted in alternating polymers’ thermal, chemical, and mechanical performances [40]; an example is the commercial polydimethylsiloxane/divinylbenzene SPME coating, which was prepared, based on the cross-linking capability of the divinylbenzene to enhance its performance [37].

In this study, a copolymer coating SPME fiber was proposed using 1-vinyl-3-butylimidazolium tetrafluoroborate (VBIMBF_4_) and ethylene glycol dimethacrylate (EGDMA) as the functional monomer and cross-linker respectively, and were consolidated via free radical polymerization. The fiber coating was synthesized on a stainless-steel wire (SSW) functionalized with vinyl-containing alkoxysilane in a glass capillary mold. Factors affecting the extraction performance of the copolymer coating were explored and optimized. The property of the fiber was evaluated by extracting PAHs in aqueous samples by headspace SPME (HS-SPME), analyzed in GC (gas chromatography), and installed with FID (flame ionization detector).

## 2. Materials and Methods

### 2.1. Reagents and Materials

PAHs standards of acenaphthene (Ace), naphthalene (Nap), acenaphthylene (Acy) Anthracene (Ant) and phenanthrene (Phe) were purchased from National Standard Material Center (Beijing, China). Polyethylene glycol (PEG, average *M*n 2000), ethylene glycol dimethacrylate (EGDMA), 3-methacryloxypropyl trimethoxysilane (MPTMS), and azodiisobutyronitrile (AIBN) were obtained from Aladdin Reagent Co. Ltd. (Shanghai, China). 1-vinyl-3-butylimidazolium tetrafluoroborate (VBIMBF_4_) was purchased from Chengjie Chemical Co. Ltd. (Shanghai, China). All the other reagents (AR-grade) were used as received. All aqueous solutions use ultrapure water as the solvents.

PAH stock solution was prepared by dissolving solid powders in methanol and was diluted in a volumetric flask at a concentration of 100 mg/L and stored at 4 °C. The working solution at a concentration of 20 μg/L was prepared daily by diluting the stock solution with ultrapure water. River water was sampled from Yellow River (Zhengzhou, China) and stored at 4 °C.

### 2.2. Instruments

Instrument: Agilent 7890B gas chromatograph (Agilent, Shanghai, China); Separation column: HP-5 (Agilent Scientific, Santa Clara, CA, USA) capillary (30 m in length, 0.32 mm in inner diameter, 0.25 μm in coating thickness); instrument conditions: sample inlet temperature: 260 °C, carrier gas: nitrogen, flow rate: 40 milliliter per minute, splitless mode, column initial temperature: 50 °C, hold time: 5 min, ramp rate: 12 °C per minute, final temperature: 250 °C, hold time: 5 min.

The coating thickness and surface morphology was characterized in a scanning electron microscope (Quanta 200 SEM, FEI, Hillsboro, OR, USA). The structural information was indicated by the infrared absorption spectrum (TENSOR-27 FTIR, Bruker, Karlsruhe, Germany). Thermal stability was also studied by thermal gravimetric analysis (Hengjiu HCT-2 TGA, Beijing, China).

### 2.3. Preparation of PIL coated SPME fiber

SSW at an outer diameter of 0.12 or 0.15 mm was cut at 6 cm long. One end of the wire (approximately 3 cm in length) was ultrasonicated with acetone and ultrapure water for 10 min, respectively, to remove any contaminants. Then, the wire was etched by aqua regia (hydrochloric acid: nitric acid = 3:1) for 1 min, thoroughly washed with ultrapure water, and dried naturally in the air. Finally, the etched part of SSW (2.0 cm) was placed in MPTMS-water-methanol solution (2:1:8, *V*/*V*/*V*) at 30 °C for 2 h, and then washed with ethanol and dried in ambient temperature.

**Figure 1:**
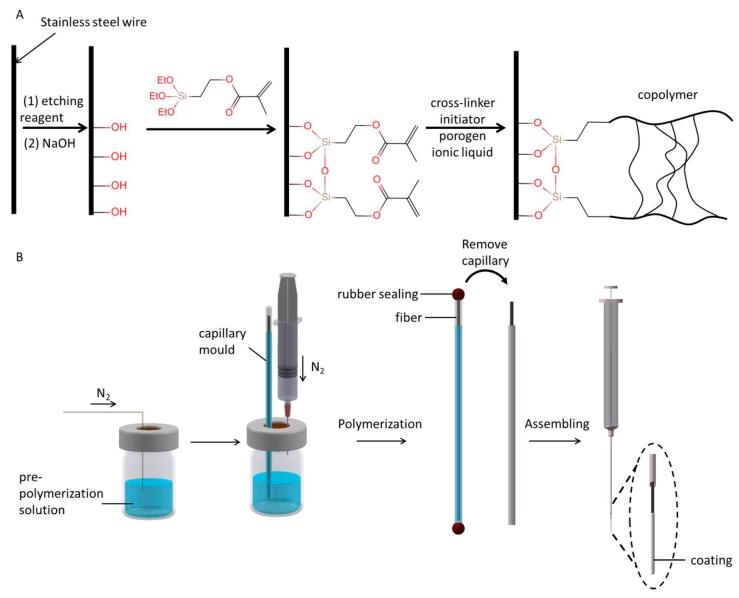
Workflow of the preparation of the copolymer coating: (**A**) Schematic diagram of the pretreatment of SSW and copolymer polymerization; (**B**) schematic diagram of the coating preparation.

The cross-linked PIL coating was prepared in a glass capillary mold according to the published procedure with some modifications [40]. The schematic preparation of the PIL coating is shown in Figure 1B. Briefly, 210 mg (1 mmol) of VBIMBF_4_, 0.95 mL (5 mmol) of EGDMA, 14.4 mg of initiator (AIBN) and 400 mg porogen agent (PEG) were dissolved in 2.5 mL of DMF as the pre-polymerization solution. The mixture was transferred into a small glass vial and sealed with an aluminum cap. Then, the pre-polymerization solution was purged with nitrogen for 5 min to remove dissolved oxygen in the solution. The vinyl-functionalized SSW was placed in a glass capillary (0.2 mm in diameter and 6 cm in length) as the mold. Then, the pre-polymerization solution was pressed into the glass capillary by applying pressure with a syringe to the vial. After that, both ends of the glass capillary were sealed with sealing membranes (Figure 1B). The glass capillary filled with the pre-polymerization solution was placed in an oven and heated at 70 °C for 8 h. Following polymerization, the polymeric fibers were manually pulled out from the glass capillary mold immediately and washed with methanol/water (1:1, *V*/*V*) to remove any residual monomers, cross-linkers, initiators, or porogen agents. The surplus part was peeled off to leave a coating length of 20 mm on the wire. Finally, the fiber was assembled onto a 5 μL syringe as the SPME device. The fiber was conditioned in the GC injector at 260 °C under nitrogen until the baseline of the chromatograph was stable.

### 2.4. Headspace SPME Procedures

The extraction experiment was performed as follows: 10 milliliters of the work solution was transferred in a 20 mL headspace vial with a magnetic stir and certain amount of salt. The vial was sealed with parafilm around the cap and was placed in the water bath at a given temperature. After 2 min of equilibration, the needle of the SPME fiber pierced through the cap. The fiber coating was pushed out from the needle and was exposed in the headspace of the vial. The stirring speed was maintained at 1200 rpm. After the extraction, the coating was retracted and thermally desorbed in the GC inlet at once. The fiber was conditioned in another GC inlet to remove any residues after each use or every day before the first use.

## 3. Results and Discussions

### 3.1. Preparation of the PIL Coating

#### 3.1.1. Pretreatment of the Substrates

Before the preparation of PIL coated fiber, the SSW was first treated with aqua regia to increase the total surface area, followed by soaking in NaOH to form an OH-rich surface. The purpose of treatment with MPTMS is to introduce alkenyl group on the surface of SSW that can be used as the polymerization reaction sites to enhance the overall mechanical stability of the SPME fiber (Figure 1A). After the functionalized SSW was put into the glass capillary mold filled with pre-polymerization solution and heated, polymerization reaction occurred among the cross-linkers, the monomers, the ionic liquids, and the alkenyl groups on the SSW surface, providing a robust coating that was covalently linked.

#### 3.1.2. Optimization of Polymerization Conditions

**Figure 2:**
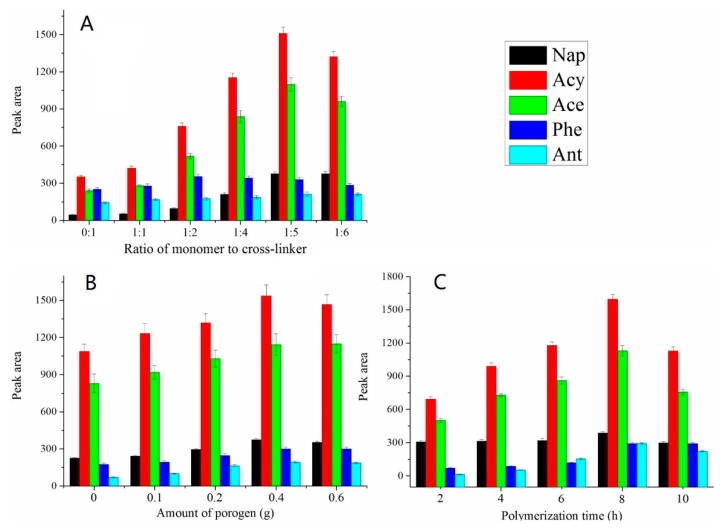
Effects of the SPME fiber preparation conditions on extraction efficiency: (**A**) molar ratio of monomer to cross-linker (amount of porogen: 0.4 g, polymerization time: 8 h), (**B**) amount of porogen (monomer: cross-linker = 1:5, polymerization time: 8 h) and (**C**) polymerization time (monomer: cross-linker = 1:5, amount of porogen: 0.4 g).

**Figure 3:**
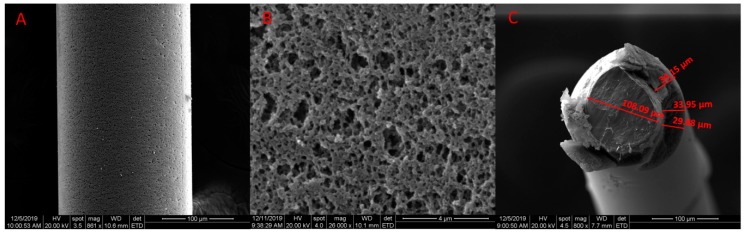
SEM images of the cross-linked PIL coating at the magnifications of (**A**) 861×; (**B**) 26000×, and (**C**) 800× cross-section view of the coating fiber.

Generally, the increase of the cross-linker will increase the crosslinking degree of the copolymer, thus leading to a stronger mechanical strength of the coating [30,33,37,41]. In this experiment, the molar ratio of VBIMBF_4_ to EGDMA was varied from 0:1 to 1:6 to investigate the influence of the cross-linker content on the extraction efficiency. As can be seen from Figure 2A, the extraction efficiency of the fibers increased with the content of the cross-linker, as expected. The cross-linking density of the co-polymer can be controlled by changing the amount of cross-linking agent, and the time and temperature of polymerization reaction [42]. A porous structure copolymer with a large specific surface area was obtained (Figure 3B), leading to an increase in the coating extraction efficiency [43,44]. However, when the mass ratio of the cross-linker to the monomer was increased to 1:6, the extraction efficiency of the coating decreased. This is most likely as a result of the reduction in the interaction between the fiber coating and the analytes as an outcome of a relative content reduction in the monomer [40,45]. Therefore, a molar ratio of 1:5 was selected, which is the same as the ratio adopted in another report [31].

Porogens play an important role in extraction performance and affect the lifetime of the fiber [46]. More porogenic agents contribute to a more porous structure, which may facilitate the diffusion of the target molecules into the solid phase, whereas it increased the brittleness of the polymer. The effect of the amount of PEG from 0 to 0.6 g on the extraction efficiency of the copolymer coating was investigated. The results (Figure 2B) showed that the highest extraction efficiency was obtained when the amount of PEG was 0.4 g. As a result, 0.4 g porogen was selected.

Polymerization time may affect the extraction performance by alternating the polymerization degree of the coating. The effect of reaction time on the extraction efficiency of the coatings was studied by during 2, 4, 6, 8, and 10 h. The results (Figure 2C) showed that the extraction efficiency increased gradually with the polymerization time. When passing 8 h, the extraction efficiency had no obvious change. Since a longer polymerization time increases the difficulty of pulling the fiber out of the mold, 8 h was selected as the optimized reaction time.

### 3.2. Characterization of the PIL coating

The surface properties and measurement of the thickness of the coating were investigated by SEM (Scanning Electron Microscope). As shown in Figure 3A,B, it can be seen that the copolymer coating exhibits a porous structure.

The thickness of the fiber coating can affect the adsorption capacity and equilibrium time of SPME. Generally, the thicker the coating, the larger the adsorption capacity, but the longer it takes to reach equilibrium. The coating with different thicknesses can be prepared by using different outer diameters of fibers and the inner diameters of the molds. In this experiment, the coating thickness is determined by using 0.12 mm (o.d.) SSW and 0.2 mm (i.d.) glass capillary. The prepared thickness of the coating was measured to be 33 μm (Figure 3A) in SEM. The slight decrease in the coating thickness might be an outcome of shrinkage after the polymerization in addition to the fiber and mold diameter errors.

Figure S1 (Supplementary Materials) shows the FT-IR spectrum of IL monomer, cross-linker, and PIL copolymers, respectively. The peak at 1658 cm^−1^ in the FT-IR spectrum of IL monomer (Curve a) corresponds to C=C stretching vibration, the peak at 1568 and 1175 cm^−1^ are attributed to the C=N and C–N stretching vibration of the imidazole, the peak at 1073 cm^−1^ is attributed to the B–F stretching vibration. The peak at 1723 and 1295 cm^−1^ in the FT-IR spectrum of cross-linker (Curve b) corresponds to C=O and C–O stretching vibrations, and the peak at 1638 cm^−1^ is attributed to the C=C stretching vibration [47]. As can be seen from Curve c, after the polymerization, both the disappeared absorption peaks at 1658 and 1638 cm^−1^ of the stretching vibration of C=C for IL monomer and cross-linker, and the appeared absorption band at 1729 and 1085 cm^−1^ for the stretching vibration of C=O and B–F, indicated the successful copolymerization between VEIMBF_4_ and EGDMA.

Since the SPME fiber will be frequently used under high temperatures when coupled with GC or GC–MS, the thermal stability of the coating is directly associated with its lifetime. Thermogravimetric analysis (TGA) was employed to evaluate the stability of the cross-linked PIL coating. The coating material was studied under the temperatures from 30 to 650 °C in an N2 atmosphere. The results (Figure S2) show that the as-prepared coating has excellent stability at temperatures below 290 °C, while the temperature exceeds 290 °C, the coating will decompose or volatilize, appearing as weight loss. Considering the normal operating temperature of the GC inlet, the final desorption temperature was set to 260 °C.

### 3.3. Optimization of the HS-SPME procedures

To obtain the best sensitivity, several potential factors affecting the extraction efficiency, including extraction time and temperature, ionic strength of the solutions, and the desorption time were investigated by the Control–Variate Method. 10 mL of work solution mixed with 20 µg/L of each analyte was used for all the optimization experiments. The average chromatographic peak area obtained by three repeated experiments was used to evaluate the extraction efficiency.

#### 3.3.1. Desorption Time

**Figure 4:**
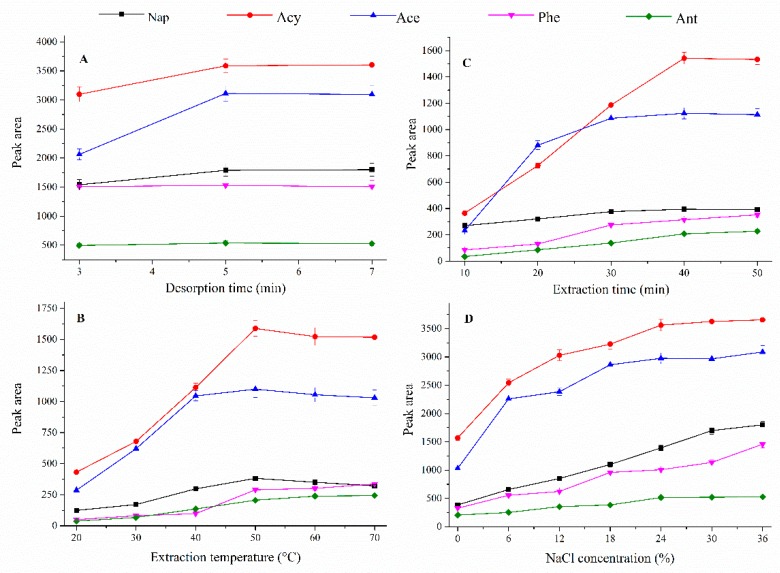
The optimization profiles of (**A**) desorption time; (**B**) extraction temperature; (**C**) extraction time; and (**D**) NaCl concentration.

A long enough desorption not only ensures a complete release of the analyte from the coating, increasing the extraction sensitivity, but avoids the influence of the analyte residues on the subsequent experiments. The effect of desorption time was investigated from 1 to 10 min under 260 °C. The results (Figure 4A) showed that all the analytes could be desorbed from the fiber within 5 min. Considering the removal of possible residues, the final desorption time was selected at 7 min.

#### 3.3.2. Extraction Temperature

Generally, increasing the temperature can accelerate the mass transfer of analyte from the work solution to the headspace, shortening the required equilibrium time. However, it alternates the distribution coefficient of the analyte between the gas phase in the headspace and the solid phase of the coating, which may reduce the extraction capability. Herein, the extraction temperature ranged from 30 to 70 °C. As seen in Figure 4B, the highest peak areas of analytes were reached at 50 °C, except Phe and Ant. To obtain the maximum total extraction capacity, 50 °C was chosen as the optimized extraction temperature for the following experiments.

#### 3.3.3. Extraction Time

Extraction time profiles were obtained at different times, ranging from 10 to 50 min at 50 °C. As can be seen from Figure 4C, the extraction efficiency gradually increase with the extraction time. For most of the analytes, the equilibrium state was reached after 40 min. Therefore, the following experiments were extracted for 40 min.

#### 3.3.4. Ionic Strength

Adding salt to the aqueous solution would affect the extraction efficiency by decreasing the solubility of the analyte in aqueous solution or increasing the viscosity of the solution. In this experiment, the effect of ionic strength was explored by varying the NaCl concentration from 0 to 36% (*W*/*V*) as 36% of the NaCl, which is the saturation concentration at 50 °C. As can be seen from Figure 4D, the extraction efficiencies of all the analytes grow with the NaCl concentration, which was in agreement with the results of other reports [48,49]. Finally, 36% was taken as the optimal salt concentration for the following experiments.

#### 3.3.5. Direct SPME and Headspace SPME

SPME is usually performed in two modes, headspace (HS) and direct immersion (DI). The choice of the extraction model depends on the characteristics of the target analyte and sample matrix. Generally, the DI mode can be applied for compounds with low volatilities in a simple matrix. If the analytes have high volatilities and the matrix of the sample is complex or in solid form, the HS mode is preferred [28]. To verify the extraction efficiency of PIL coating for PAH compounds in DI and HS modes, 10 mL standard solution, with a concentration of 20 μg/L, was extracted at 30 and 50 °C with DI and HS modes, respectively. The results (Figure S3) showed that the overall extraction efficiency of PAHs in the HS mode performs better than that in the DI mode, as expected. The difference is even more obvious at low extraction temperatures. Herein, HS-SPME is used throughout this paper to analyze PAHs in aqueous samples.

#### 3.3.6. Extraction Characteristics of Copolymer Coating

To study the extraction characteristics of the copolymers coating, non-polar compounds with and without benzene rings (PAHs, toluene, and tridecane), and polar organic compounds with and without benzene rings (benzyl alcohol and dodecanol) were selected as target analytes. The enrichment factor (EF) was used to evaluate the extraction characteristics of the coatings. It is defined as the ratio of the chromatographic peak area response to the SPME extraction of 10 mL of standard solution (10 µg/L) to that with direct injection of 1 µL of standard solution (10 mg/L) [50]. HS-SPME experiments were performed under respective optimized conditions [51,52,53,54]. As can be seen from Table S1 (Supplementary Materials), the fiber has a high enrichment efficiency for two-ring PAHs, long-chain aliphatic alcohols and alkanes, and the lowest enrichment effect was obtained for benzyl alcohol because benzyl alcohol is the most polar analyte. Based on these results, we can infer that the influence extraction efficiency of target analytes can be attributed to a combination of multiple interactions including π–π conjugation, hydrophobic force, and the molecular sieve effect [55,56]. Furthermore, the PIL copolymer coating fiber can also be used for the enrichment of dodecanol and tridecane in water samples.

### 3.4. Analytical performance of the developed method for PAHs

In order to evaluate the performance of the developed method, important parameters, including linear ranges (LRs), limit of detections (LODs), and repeatability were investigated under optimized conditions. The results are shown in Table S2 (Supplementary Materials). LRs for analytes were 0.1–100 μg/L with a correlation coefficient (r) ranging from 0.9994 to 0.9999. LODs (S/N=3) were in the range of 0.003–0.026 μg/L. Single-fiber and fiber-to-fiber repeatability were investigated by five replicate extractions under the same conditions. The relative standard deviations (RSDs) were in the range of 4.8%–11.9% and 5.1%–9.8%, respectively.

The performance of the as-prepared fiber was also compared with other PIL coatings under the optimal extraction conditions. PAHs compounds in water were analyzed in either SPME–GC–FID or SPME–GC–MS. A detailed comparison can be found in Table 1. It can be seen that the performance of the method in this paper is similar or succeeds the reported results in terms of LODs and LRs, indicating the high sensitivity of this newly developed fiber and its potential application in extracting or enriching PAHs in water.

### 3.5. Application to Real Samples

To confirm the practicability of the proposed device and the method, the PIL coating fiber was applied to determine the contents of PAHs in the river water samples. No PAH compounds listed in Table S1 (Supplementary Materials) were detected. To further investigate the effect of sample matrix on the extraction performance, relative recoveries were studied by spiking the river water samples with standard PAH mixtures. The final concentrations were kept at 5 and 50 μg/L, respectively. The chromatograms of the sample and the spiked sample are found in Figure S4 (Supplementary Materials). As shown in Table 2, the recoveries (average values of five measurements) ranged from 84.2% to 109.2%. These results indicate that the complex sample matrix has no significant effect on the extraction performance of the PIL fiber, which proves that the proposed method has potential in practical applications.

## 4. Conclusions

In the present work, a novel polyionic liquid copolymer was prepared via free radical polymerization and was employed as an SPME fiber adsorbent. The in-mold polymerization approach uses negligible solvents and enables the high-throughput production of SPME fibers with good repeatability. The coating thickness is arbitrarily determined by changing the inner diameter of the glass capillary and the outer diameter of the stainless-steel wire. The prepared fiber exhibited high mechanical strength and thermal stability due to the cross-linking structure and immobilization of the coating on the stainless-steel wire surface by the covalent bond. Compared with commercial PDMS fibers, the proposed SPME fiber shows lower detection limits, higher extraction efficiency, and longer lifetime. The satisfactory results in the spiked river water indicate its potential applications in detecting PAHs in foods or soils that have more complicated matrices.

## Figures and Tables

**Table 1:** Comparison of the developed method with other reported methods for the determination of PAHs in water.

Method	Coating/Thickness	Analytes	LRs (μg/L)	LODs (μg/L)	Ref.
HS-SPME GC-FID	PIL-Benzyl/1 μm	Nap	0.05–20	0.02	[25]
		Acy	0.1–20	0.04	
		Phe	0.1–20	0.01	
		Ant	0.1–20	0.01	
DI-SPME GC-FID	Poly(VBHDIm^+^NTf_2_^−^)/12 μm	Nap	0.1–20	0.031	[26]
		Acy	0.1–20	0.006	
		Phe	0.5–20	0.007	
		Ant	2–20	0.052	
DI-SPME GC-MS	Poly(VBIm^+^Cl^−^)/19 μm	Nap	0.5–20	0.12	[27]
		Ace	0.5–20	0.05	
		Acy	0.5–20	0.06	
		Phe	0.5–20	0.25	
		Ant	0.5–20	0.10	
DI-HS-SPME GC-MS	Poly[(VBIM)_2_C1_2_]_2_[NTf_2_]@[VBC_16_IM] [NTf_2_]/21 μm	Nap	0.01–10	0.003	[28]
		Acy	0.01–10	0.003	
		Ace	0.05–10	0.015	
		Phe	0.05–20	0.015	
		Ant	0.05-10	0.015	
HS-SPME GC-FID	PDMS/100 μm	Nap	0.1–100	0.15	[57]
		Acy	5–100	0.21	
		Ace	5–100	0.10	
		Phe	5–100	0.12	
		Ant	5–100	0.18	
HS-SPME GC-FID	Poly(VEIMBF_4_)@EGDMA/33 μm	Nap	0.1–100	0.009	This method
		Acy	0.1–100	0.003	
		Ace	0.1–100	0.003	
		Phe	0.1–100	0.011	
		Ant	0.5–50	0.026	

**Table 2:** The recovery and precision of the extraction performance for PAH compounds in spiked river water samples.

Analyte	Spiked Level: 5 μg/L		Spiked Level: 50 μg/L	
	Recovery (%)	RSD (%)	Recovery (%)	RSD (%)
Nap	92.1	6.3	99.6	4.9
Acy	100.6	2.8	105.1	3.4
Ace	94.2	3.6	109.2	4.2
Phe	105.6	3.0	96.0	3.9
Ant	84.2	5.2	100.2	4.5

RSD: Relative standard deviation, in percent (*n* = 5).

## Supplementary Materials

The following are available online at https://www.mdpi.com/2073-4360/12/2/292/s1, Figure S1: The infrared spectra of the monomer (a), cross-linker (b) and copolymer (c), Figure S2: TGA curve of the copolymer, Figure S3: Comparison of HS-SPME and DI-SPME extraction mode, Figure S4: Chromatograms for river water (bottom curve) and spiked river water (top curve, spiked concentration: 5 μg/L). Table S1: Extraction characteristics of copolymer coating for various analytes, Table S2: Analytical performance of GC-FID for analyzing PAHs using PIL coated fiber
