# Supplementary material for: Determination of Volatile Water Pollutants Using Cross-Linked Polymeric Ionic Liquid as Solid Phase Micro-Extraction Coatings
Source: Polymers (Basel). 2020 Feb 2;12(2):292. doi: 10.3390/polym12020292 (PMC7077427; doi:10.3390/polym12020292)
Supplement: Supplementary file 1 [file polymers-12-00292-s001.pdf]

Supporting Information

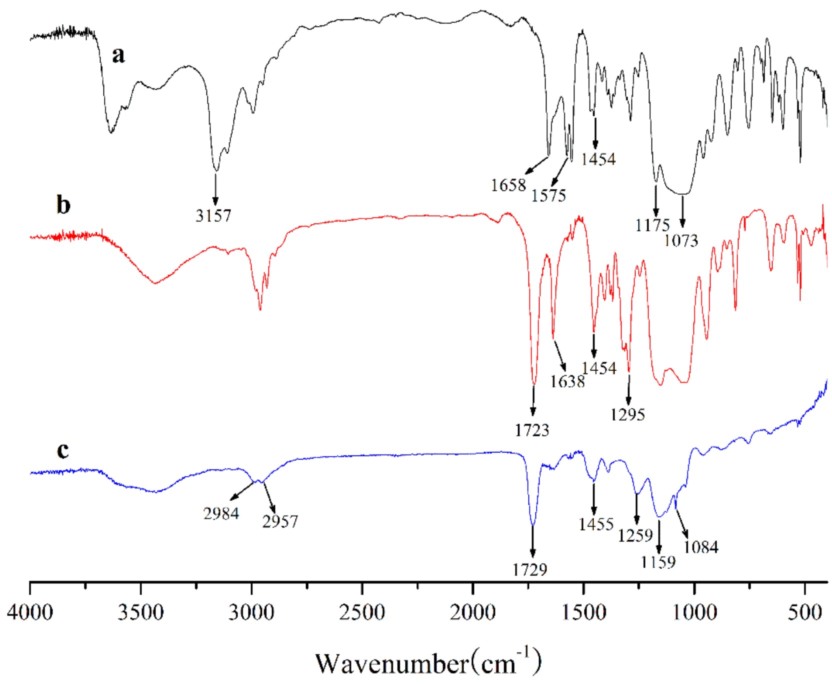

Figure S1. The infrared spectra of the monomer (a), cross-linker (b) and copolymer (c)

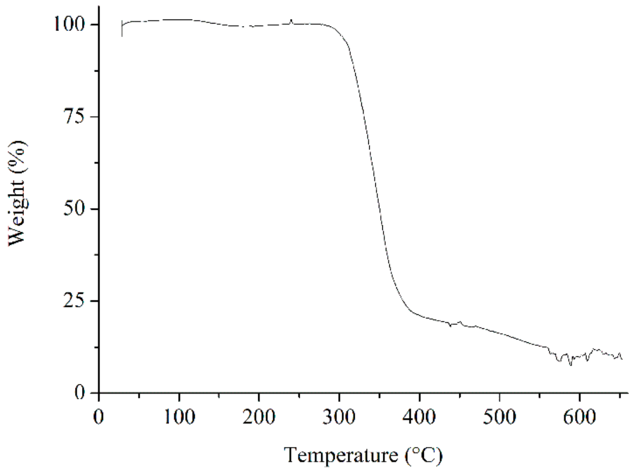

Figure S2. TGA curve of the copolymer

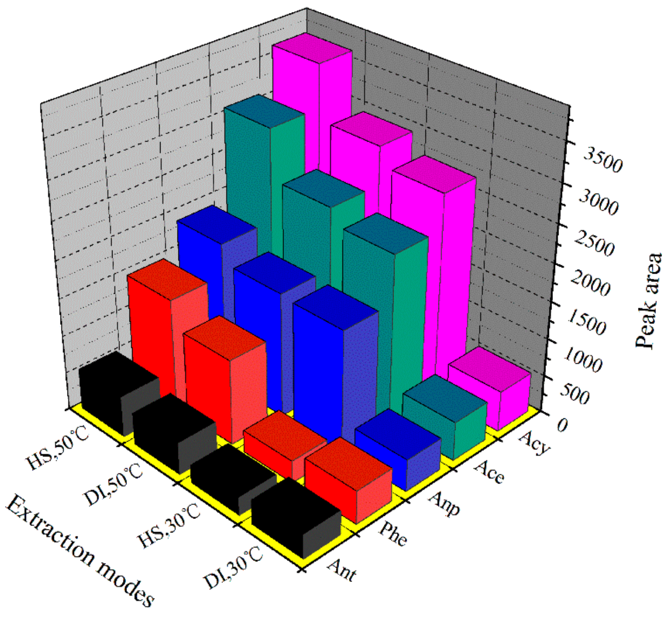

Figure S3. Comparison of HS-SPME and DI-SPME extraction mode

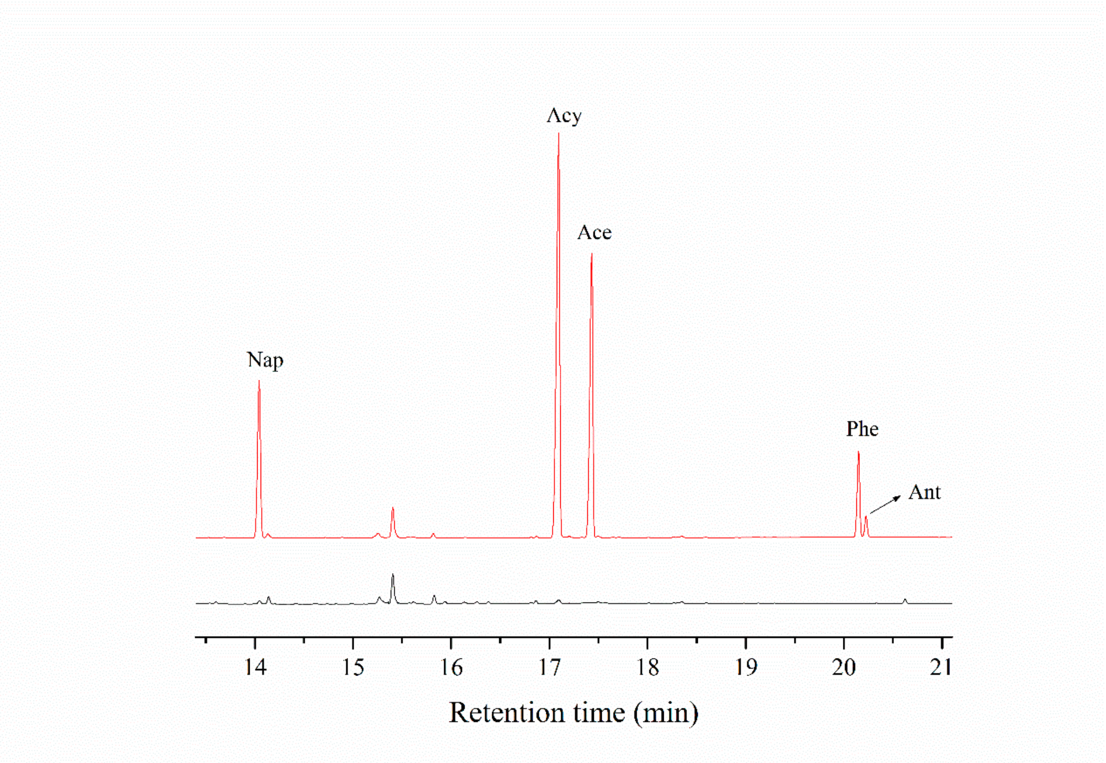

Figure S4. Chromatograms for river water (bottom curve) and spiked river water (top curve, spiked concentration: 5 μg/L).

Table S1. Extraction characteristics of copolymer coating for various analytes

Analytes	Non-polar compounds							Polar compounds	
	With Pha						Without Pha	With Pha	Without Pha
	Nap	Acy	Ace	Phe	Ant	toluene	tridecane	Benzyl alcohol	dodecanol
**EFs**	20993	24056	13336	7193	2980	3084	36297	168	17064

[a] Ph:benzene ring

Table S2. Analytical performance of GC-FID for analyzing PAHs using PIL coated fiber

Analyte	Regression Equation	LRs(μg/L)	r	LODs(μg/L)	Repeatability (RSDs,%)	
					Single-fiber(n=5)	Fiber to fiber(n=5)
Nap	y = 38.02 x - 30.05	0.1-100	0.9994	0.009	6.1	5.6
Acy	y = 64.29 x + 36.61	0.1-100	0.9990	0.003	11.1	8.7
Ace	y = 28.79 x + 40.01	0.1-100	0.9987	0.003	4.8	5.1
Phe	y = 11.02 x + 3.53	0.1-100	0.9995	0.011	11.9	9.8
Ant	y = 11.64 x - 5.55	0.5-50	0.9999	0.026	8.9	7.5
